# Study on the Optimization of Cu-Zn-Sn-O to Prepare Cu_2_ZnSnS_4_ Thin Film via a Nano Ink Coating Method

**DOI:** 10.3389/fchem.2021.675642

**Published:** 2021-05-28

**Authors:** Qian Li, Jinpeng Hu, Yaru Cui, Juan Wang, Jinjing Du, Miao Wang, Yu Hao, Tong Shen, Lizhen Duan, Simin Wang, Ke Sun

**Affiliations:** School of Metallurgical Engineering, Xi'an University of Architecture and Technology, Xi'an, China

**Keywords:** Cu-Zn-Sn-O(CZTO), precursor, coprecipitation, Cu_2_ZnSnS_4_(CZTS) thin film, nano ink, band gap

## Abstract

To reduce the formation of the impurity phase, a buffer volume can be used to expands and smooths the surface of Cu_2_ZnSnS_4_(CZTS) thin film. In this study, a Cu-Zn-Sn-O(CZTO) precursor was synthesized through the process of coprecipitation-calcination-ball milling-spin coating. The influence of pH, temperature, and PVP on the constituent of hydroxides was investigated in the process of coprecipitation. Cu-Zn-Sn-O with appropriate compositions could be obtained by regulating the temperature and preservation time of the calcination stage. After ball milling to form a nano ink, and then spin coating, SEM images proved the generation of CZTO precursors, which effectively promoted the formation of Cu_2_ZnSnS_4_ thin films. Finally, the phase, microstructure, chemical composition, and optical properties of the Cu_2_ZnSnS_4_ thin films prepared by sulfurized annealing CZTO precursors were characterized by EDX, XRD, Raman, FESEM, Hall effect, and UV methods. The prepared CZTS thin film demonstrated a band gap of 1.30 eV, which was suitable for improving the performance of CZTS thin film solar cells.

## Introduction

In recent years, due to the exhaustion of fossil energy, many scientists have begun exploring renewable energy (Sharma et al., 2019; Kou et al., 2020; Liu et al., 2021; Nie et al., 2021), among which solar energy is a key energy source, and leading to a wide range of research (Xiao et al., 2018; Chen et al., 2019). As one of the main thin film solar cell materials, CIGS has achieved great success for its high device efficiency (Chirila et al., 2013). Up to now, the highest recorded efficiency of CIGS was reported by Philip Jackson, reaching an astonishing 22.6% (Jackson et al., 2016). However, the disadvantages of this material are becoming increasingly apparent. The use of the rare elements In and Ga and toxic element Cd has greatly restricted the large-scale application of CIGS solar cells (Tumbul et al., 2019; Plass et al., 2020). Therefore, CIGS solar cells failed to meet the requirement of sustainable development.

CZTS has a stable perovskite structure with a suitable band gap of 1.0–1.5 eV and a high absorption coefficient of 10^4^/cm. The preparation of this thin film material using non-toxic and abundant elements makes it a suitable candidate to replace CIGS as a thin film solar cell (Washio et al., 2012; Azmi et al., 2021; Kaur et al., 2021; Li X. M. et al., 2021; Ramirez-Ceja et al., 2021). A variety of experimental methods to prepare CZTS thin film materials have been developed, such as electrospinning (Bi et al., 2015; Mu et al., 2015), nano ink method (Altowairqi et al., 2019; Liu et al., 2020), sol-gel (Dong et al., 2017; Jacob et al., 2021), sputtering (Lu et al., 2018; Pandharkar et al., 2021) and electrodeposition (Khattak et al., 2019; Demir, 2021), and so forth. Kim et al. mixed CZTS ink with 2-methoxyethanol, with the highest efficiency of 8.17% (Kim et al., 2014). After an in-depth study, Wang et al. introduced the Se element to obtain CZTSSe thin film solar cells, and finally achieved 12.6% high efficiency (Wang et al., 2014). However, the existing recorded efficiency of the device is still far lower than the theoretical efficiency of 32.2% (Guo et al., 2009).

It was found that the CZTS was generated by using CZT as a precursor that produces obvious impurity phases such as Cu_x_S, which increases the surface defects of CZTS thin films, thus affecting the short-circuit current and open-circuit voltage of CZTS solar cells, and ultimately reducing device efficiency (Yoo et al., 2012; Exarhos et al., 2019). A new precursor, Cu-Zn-Sn-O(CZTO), could avoid the sudden volume expansion and prepare smooth CZTS thin films, which effectively improve the morphology of thin films, and an oxygen atom and can replaced by an S atom from CZTO to CZTS (Ishino et al., 2013; Liu et al., 2018; Li Y. B. et al., 2021). Using CZT composite oxides as a precursor, Tang et al. generated CZTS thin films by sulfurization of CZTO. By controlling the chemical co-precipitation conditions, the material was obtained with proper particle size and composition, and the obtained CZTS solar cell had a band gap of 1.35 eV (Tang et al., 2013). Ryo Ishino et al. introduced oxygen atoms diffused into the precursors, which greatly improved the surface density of the thin films compared to precursors without oxygen. Moreover, oxygen could be replaced by sulfur during the sulfurization process (Ishino et al., 2013). Li et al. prepared oxygen containing precursors by sputtering ZnO, SnO_2_, and Cu targets. The research proved that the stable structure of SnO_2_ reduces the loss of Sn, and Sn^4+^ ion and could inhibit the production of Sn^2+^ ion, which was conducive to the formation of highly crystalline thin films with a uniform surface. Finally, CZTS solar cells were prepared with an efficiency of 5.08% (Li et al., 2020). Yuta Nakayasu et al. demonstrated that when CZTS films were prepared using Cu-Zn-Sn-O as the precursor system, the sulfurization was complete and the diffusion of elements into oxides was faster than in alloys. Combining with SCF sulfurization and Cu-Zn-Sn-O precursors, the prepared CZTS thin films consisted of Cu-poor and Zn-rich composition, and the band gap of 1.38 eV was consistent with the theoretical band gap value (Nakayasu et al., 2017). In summary, CZTO precursors have a great advantage in preparing CZTS thin films such as reducing secondary phase generation, improving the quality of CZTS thin films, and alleviating volume expansion, which are ideal precursors for the preparation of CZTS thin films.

In this paper, Cu-Zn-Sn-O(CZTO) precursor films were prepared by spin coating on Mo-based substrates with CZTO nano ink synthesized by calcination and ball milling of metal hydroxides. The study revealed the effects of pH, co-precipitation temperature, and PVP content on the composition and particle size of metal hydroxides, and determined the optimization conditions of the coprecipitation process. The research also focused on the change of calcining temperature and holding time on the particle size of Cu-Zn-Sn-O obtained by calcination of metal hydroxides, and further investigated the morphology of the CZTO thin films obtained by spin coating. Finally, the CZTS thin films were obtained through sulfurization to replace O atoms in CZTO with S atoms.

## Experimental

**Figure 1:**
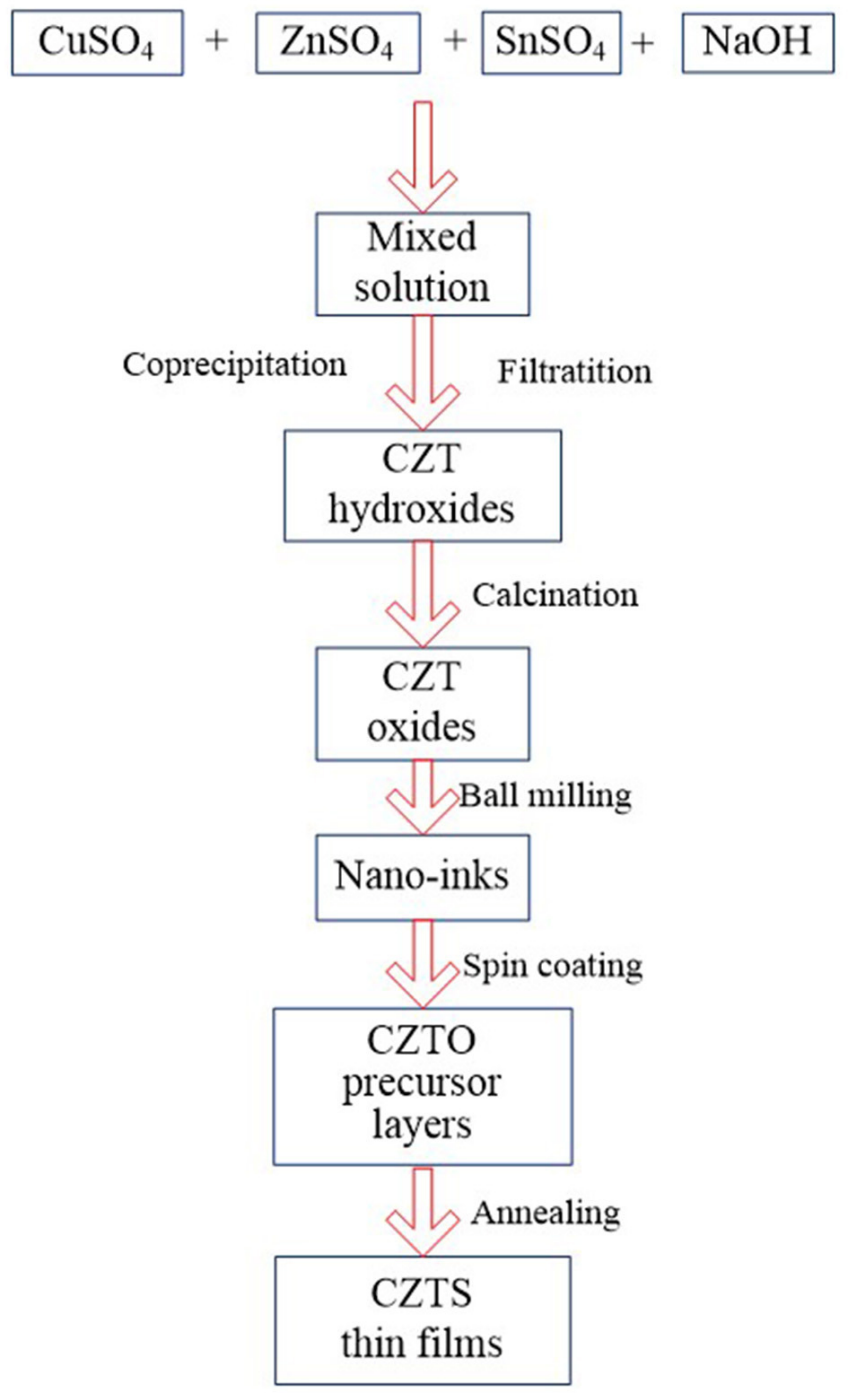
Processes for preparing Cu_2_ZnSnS_4_ thin films.

Figure 1 shows the flow chart of oxide precursor layers prepared following the process of coprecipitation-calcination-ball milling-spin coating. The fabrication process of the oxide precursor layer had a great influence on the preparation of CZTS thin films which were attained by sulfurization of CZTO precursor layers.

### Synthesis of CZT Hydroxides

According to the theoretical proportion of CZTS, the mole ratio solution of copper sulfate: zinc sulfate: stannous sulfate: sodium hydroxide was 2:1:1:4 blended homogenously. The mixture solution and sodium hydroxide were simultaneously dropped into a mass fraction of PVP solution to prepare CZT hydroxides. The influence of pH, temperature, and PVP on the constituents of the hydroxides with filtering, washing, and vacuum-drying at 60°C were investigated.

### Synthesis of CZTO Thin Films

The prepared CZT hydroxides were calcined in a tube furnace under an atmosphere of oxygen to obtain CZTO powder. In the process of calcination, the effects of temperature and preservation time on the composition of CZTO were subsequently studied. After that, CZTO was dispersed into ethanol with 1% methylcellulose and 1% PVP, and subsequently ball milled for 6 h, with a rotation speed of 600 r/min to form the homogeneous nano ink. The oxides precursor named Cu-Zn-Sn-O(CZTO) was gained by spin coating on the clean Mo-based substrate with the cycle of low speed of 800 r/min (time 6 s)—high speed of 2700 r/min (time 6 s) coating for 12 times, and then dried at 60°C overnight.

### Synthesis of CZTS Thin Films

In a typical procedure, the excess sulfur powder and CZTO thin film were placed on the upstream side and downstream side of the tube furnace, then sulfide at 580°C for 1 h under a reduced atmosphere of argon at a heating rate of 10°C/min to fabricate CZTS thin films.

### Characterization

The constituents of metal hydroxides and Cu-Zn-Sn-O were determined using chemical methods. The sizes of hydroxides and Cu-Zn-Sn-O were measured by Zeta Sizer (Zano-ZS). The surface and cross-section views of prepared CZTO precursor layers and the surface and cross-sectional morphology of the CZTS thin films were conducted by FESEM (TESCAN MIRA3 LMU). The compositions of precursor layers and CZTS thin films were determined by EDX (Oxford X-Max20). Phase information was conducted from the XRD (λ = 1.54 Å, 40 kV acceleration voltage). The further phase differentiation was tested with Raman (LABRAM-HR). Carrier concentration, hall mobility, and resistivity of CZTS thin films were measured by Hall effect measurements (HMS-3000/0.55T). The transmittance spectrum of CZTS thin films was characterized by a UV-Vis spectrophotometer (HITACHI U-4100) in wavelength coverage of 300–900 nm.

## Results and Discussion

### The Effect of pH on the Composition of Hydroxides

**Figure 2:**
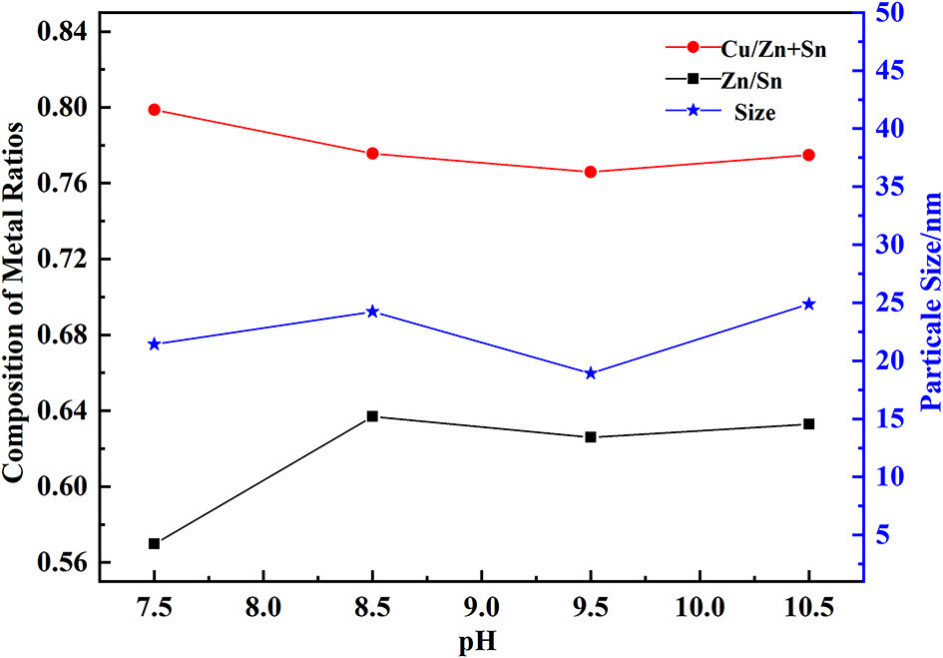
Effect of pH on the composition of hydroxides.

Three kinds of metal salt solutions and NaOH solution were added into the bottom and kept at an appropriate pH, thus OH^−^ and M^n+^ ions generating into M(OH)_n_. The value of pH had a great influence on the composition of hydroxides, as shown in Figure 2 with the temperature at 30°C, and PVP mass fraction of 0.5%. It was observed that Cu/(Zn+Sn) decreased from 0.8 to 0.77, but Zn/Sn increased from 0.58 to 0.68 with a pH range of 7.5–10.5. We used Cu/(Zn+Sn) and Zn/Sn which had a great influence on the formation of CZTS films (Chen et al., 2015; Sripan et al., 2017). At pH = 9.5, the particle size of hydroxide was 17.3 nm which would be in favor of precursor formation. When pH went to a low value, the process of Cu-Zn-Sn coprecipitation was not completed. On the contrary, the coprecipitation was prone to dehydrate with a high value of pH, meanwhile, the homogeneity of hydroxide composite particles was destroyed and coprecipitation dissolution occurred. The surface area of newly formed hydroxide particles was larger than stationary particles, so its solubility was higher. The coprecipitation dissolution could cause the constitution of the product to deviate from stationary particles.

### The Effect of Temperature on the Composition of Hydroxides

**Figure 3:**
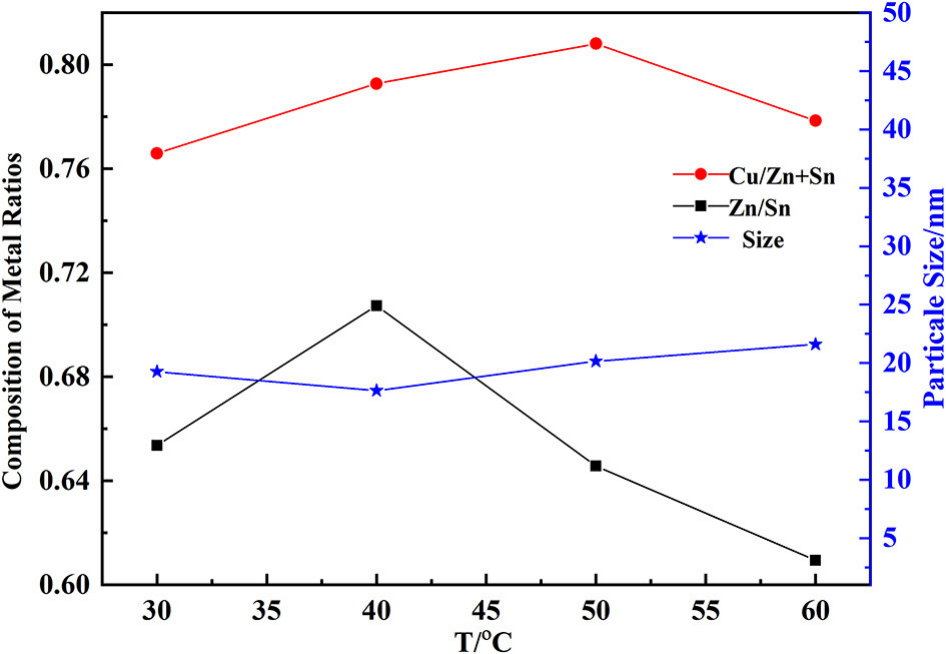
Effect of temperature on the composition of hydroxides.

When the temperature increased, both Cu/(Zn+Sn) and Zn/Sn decreased firstly and increased later (Figure 3 with pH of 9.5, the mass fraction of PVP of 0.5%). This may be due to the change of solubility of hydroxide. Cu/(Zn+Sn) and Zn/Sn were 0.79 and 0.70 at 40°C with a particle size of 17.6 nm which would be in favor of precursor formation (Tang et al., 2013). The hydroxide particle size became a little larger with increasing temperature, but this trend was not obvious. The forming process of coprecipitation could be divided into the nucleation stage and growth stage of nucleation. When the speed of nucleation formation was much faster, a large number of crystal nuclei would be formed in the solution, generating smaller particles. On the contrary, larger particles were produced. The energy of hydrolysis reaction was more and more abundant with increasing temperature, and the particles would be impacted, intensely accelerating the growth speed to produce larger particles.

### The Effect of PVP on the Composition of Hydroxides

**Figure 4:**
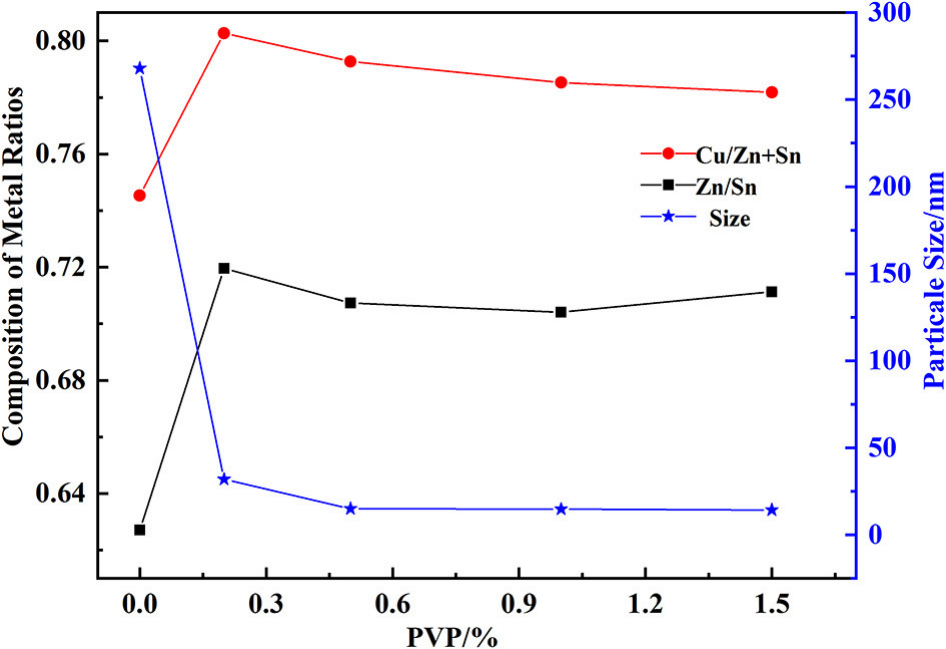
Effect of PVP on the composition of hydroxides.

The effect of PVP on the constituent of hydroxides was obtained in Figure 4 with a pH of 9.5, at a temperature of 40°C. Cu/(Zn+Sn), Zn/Sn, and the particle size of hydroxide particles had little change with the mass fraction of PVP from 0.2 to 1.5%. Without PVP, Cu/(Zn+Sn) and Zn/Sn had the lowest value, and the particle size of hydroxide kept the largest value of 268 nm. The function of PVP was to prevent agglomeration of hydroxide particles, when the mass fraction of PVP was low or not added, the dispersion was not completed, unable to effectively prevent the agglomeration of hydroxide particles leading to the formation of large particles.

### The Effect of Calcination Temperature on the Comp'osition of Cu-Zn-Sn-O

**Figure 5:**
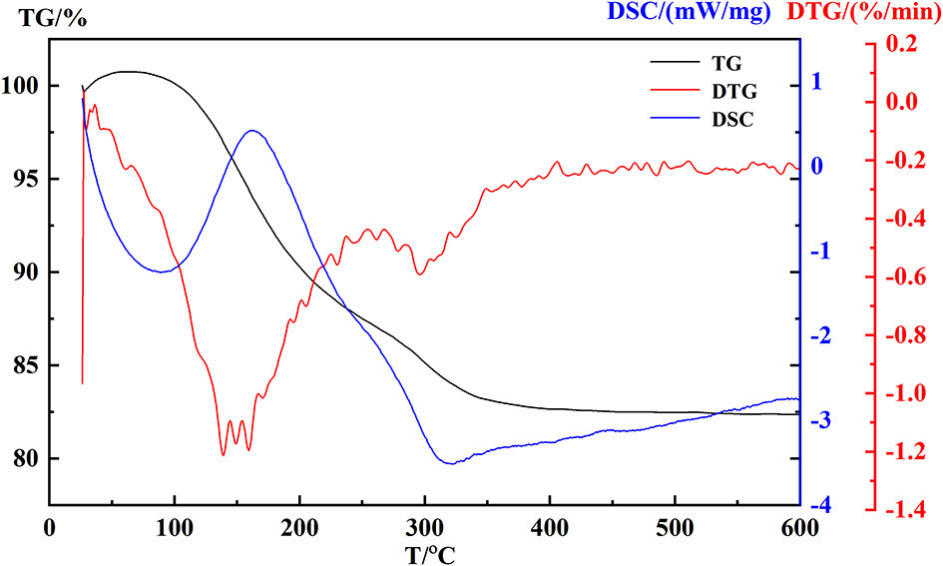
TG–DTG-DSC profiles of hydroxides.

The behavior of coprecipitation of hydroxides was chartered by TG-DTG-DSC in Figure 5. A weight loss of about 13.3% occurred at 112–222°C with a broadening exothermic region, which was attributed to the physical adsorption of water loss of hydroxides. Corresponding to the temperature region 240–550°C, a little weight loss (~4.82%) indicated that the process of removal reaction of OH bond occurred.

**Figure 6:**
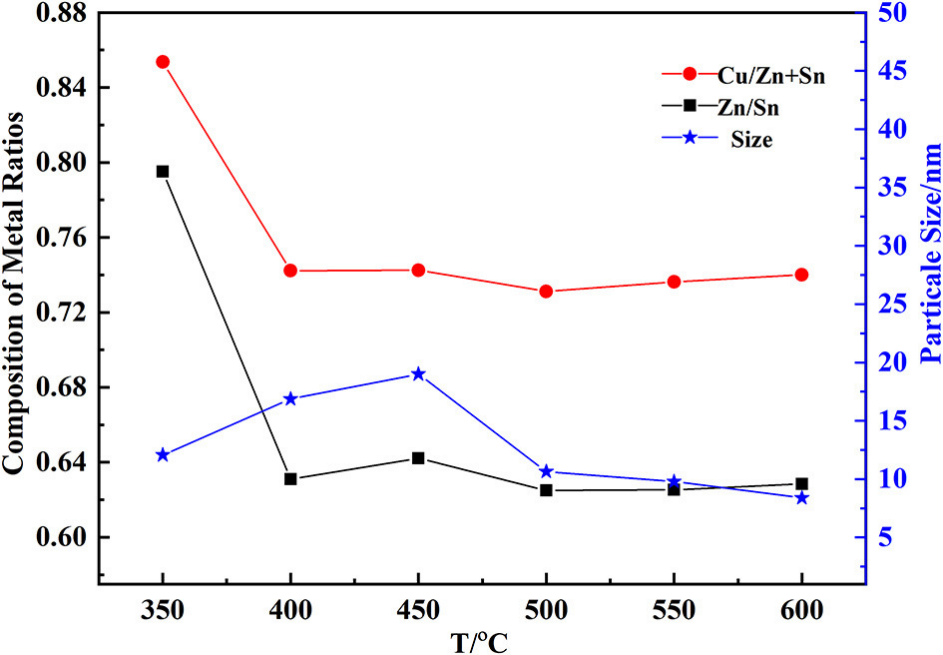
Effect of calcination temperature on the composition of CZTO.

The constituent of CZTO affected the efficiency of CZTS thin film. Cu/(Zn+Sn) and Zn/Sn could be suitable for the formation of CZTS thin film. From Figure 6, the calcination temperature made a great influence on the composition of CZTO. A few 10 nanometers of CZTO particles would be advantageous to the dense formation of CZTS. The size decreased when the calcination temperature increased above 450°C, maybe because the growth rate of particles was lower. In addition, Cu/(Zn+Sn) and Zn/Sn remained almost unchanged in the range of 400–600°C. At 550°C, the CZTO had an average particle size of 9 nm, which was the suitable temperature for the synthesis of CZTO thin film.

### The Effect of Preservation Time on the Composition of Cu-Zn-Sn-O

**Figure 7:**
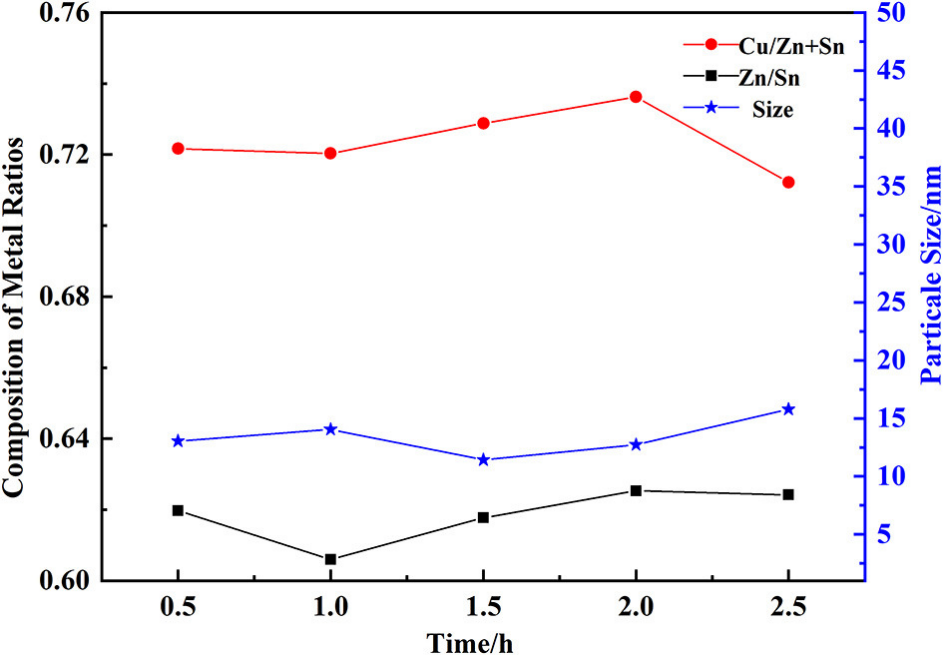
Effect of preservation time on the composition of CZTO.

After the high temperature of calcination, hydroxide particles were formed into corresponding oxide crystal particles, and the process of calcination was an important step to prepare the suitable particle size and the molar ratio of CZTO showed in Figure 7. The particle size of CZTO had an average size of 10 nm in the preservation time range of 0.5–2.5 h. The growth rate of particles was slower, extending preservation time, which may be explained by the fact that the other oxides between the same oxides were blocked, restricting the growth of CZTO particles. Therefore, 2 h of preservation time was selected as the optimal preservation time.

### SEM Surface Morphologies for CZTO Precursor Thin Films

The fabrication of CZTO precursor thin films was conducted as follows: 1.5 g CZTO were added in 30 ml ethanol with 1% methylcellulose and 1% PVP, then ball milled for 6 h, 600 r/min to formulate uniform nano ink. To avoid the possible question of crack, nano ink was spin coated on clean Mo-sputtered substrates, which were firstly pre-dried at 40°C. By controlling different temperatures with the range of 70–270°C the desired precursor could be obtained.

**Figure 8:**
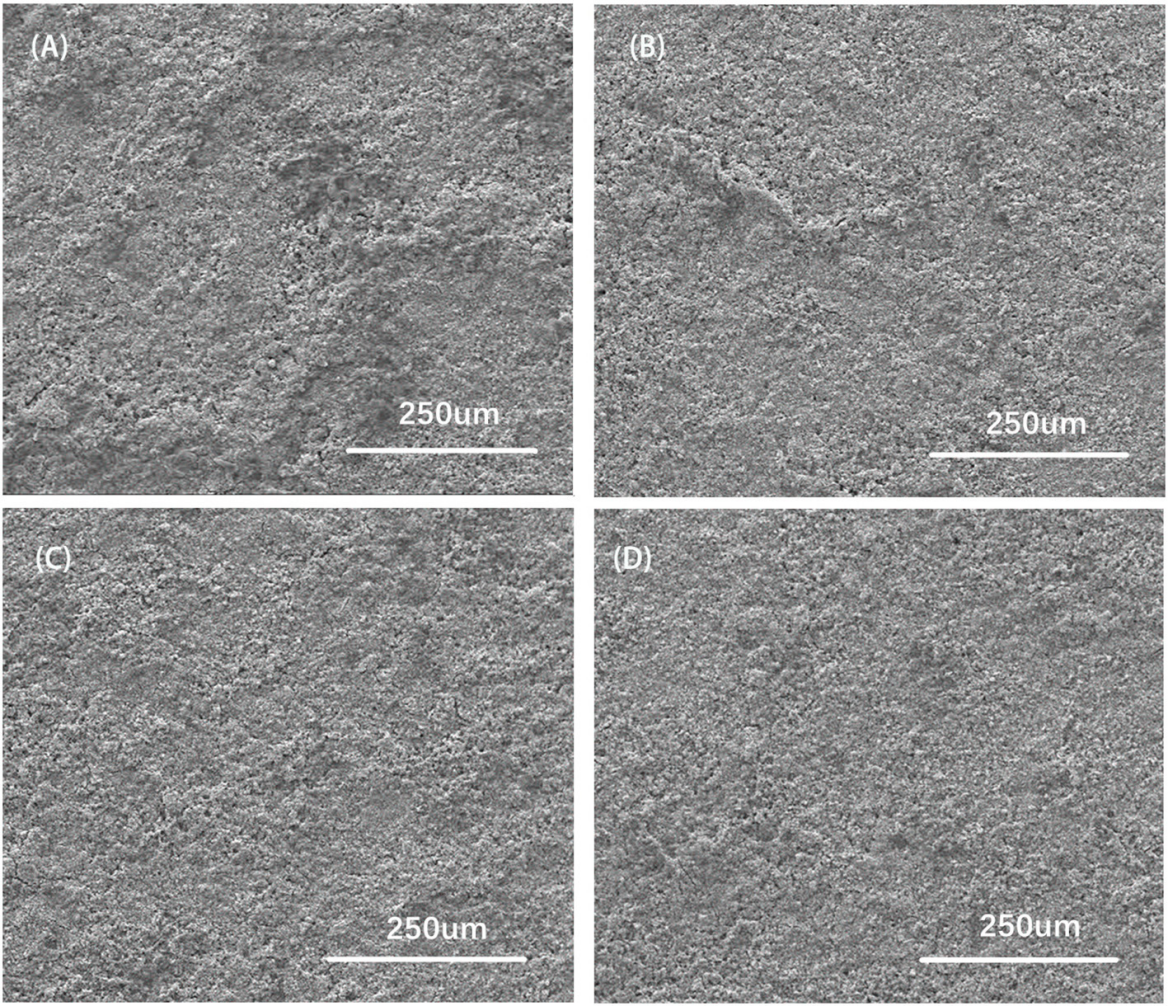
Surface SEM views of CZTO precursor thin films at different substrate temperatures **(A)** 70°C; **(B)** 140°C; **(C)** 210°C; **(D)** 270°Cs.

The surface of SEM images characterized by FESEM for CZTO precursors is presented in Figure 8. It was noted that the surface roughness of the CZTO precursor layer decreased when the substrate temperature went up from 70 to 270°C. The CZTO thin films coated at low temperature showed uneven particles as shown in Figures 8A,B. As the temperature increased, the surface roughness of CZTO decreased. When the temperature arrived at 270°C, the surface of CZTO thin films exhibited smooth morphology, the roughness decreased obviously, which was conducive to improving the performance of CZTS thin films. Accordingly, it was possible to get a smoother surface of CZTO precursors, reducing the coating times or prolonging the pre-drying time of Mo-based glass substrates.

**Figure 9:**
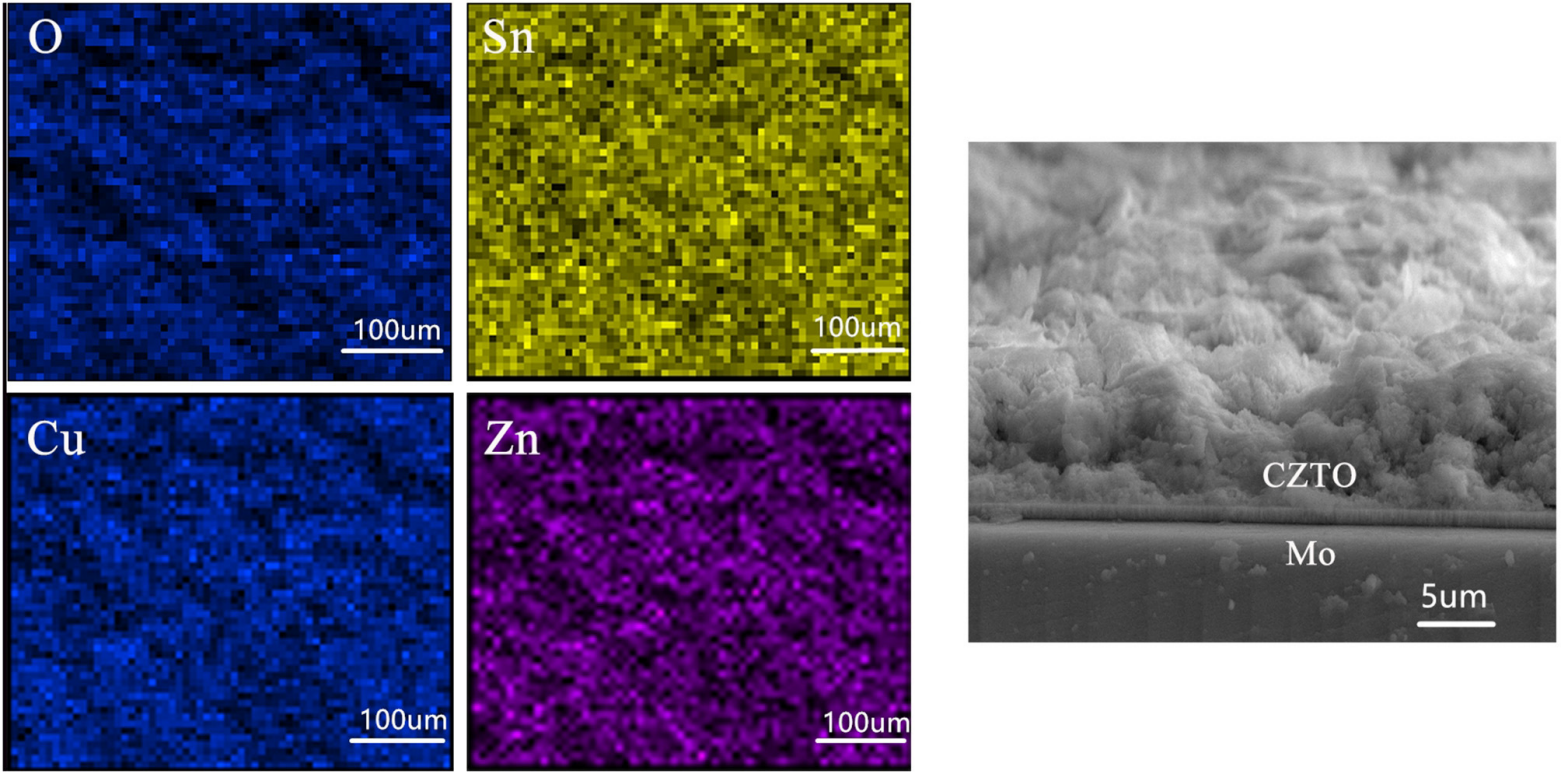
Surface elemental composition and cross-sectional views of the CZTO precursor.

The surface of elemental composition and cross-sectional SEM views of CZTO precursor after spin coating at 270°C are displayed in Figure 9. The elements of Cu, Zn, Sn, and O were observed uniformly without element enrichment and the CZTO precursor named CZTO was smooth. This could be beneficial for the performance of CZTS solar cells owing to the grain boundaries and surface defects of improvement.

### Characterization of CZTS Thin Films

To indicate the feasibility of this method, the prepared CZTO precursor was sulfurized at 580°C. The compositions of the element in precursor and Cu_2_ZnSnS_4_ thin film were obtained, as shown in Table 1. Compared to the CZTO precursor, the Cu/(Zn+Sn) ratio of Cu_2_ZnSnS_4_ thin film was almost unchanged and remained within a reasonable range. Meanwhile, the ratio of Zn/Sn increased significantly from CZTO to CZTS, which could help to suppress the self-compensating defects, creating more vacancies, and increasing the concentration of shallow receptors (Chen et al., 2019; Sun et al., 2019). In addition, the oxygen content in CZTO was almost the same as the sulfur content in CZTS, indicating that O was completely replaced by S (Liu et al., 2018).

**Table 1:** Compositions of precursor and Cu_2_ZnSnS_4_ by EDS.

	**Cu(at%)**	**Zn(at%)**	**Sn(at%)**	**O**	**S**	**Cu/(Zn+Sn)**	**Zn/Sn**
Material	50	25	25	-	-		
Precursor	10.38	4.47	5.66	53.57	-	0.94	0.79
Cu_2_ZnSnS_4_	22.95	11.38	12.51	-	53.16	0.96	0.91

**Figure 10:**
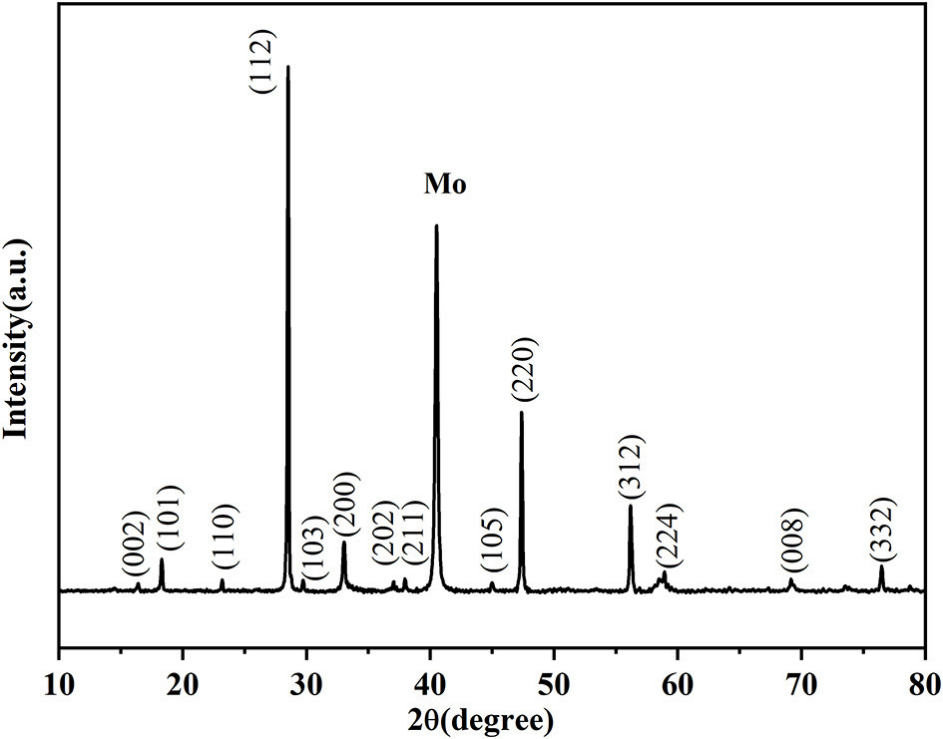
XRD patterns of Cu_2_ZnSnS_4_ thin film annealed at 580°C.

The XRD patterns of CZTS thin film are displayed in Figure 10. The sharp diffraction peaks matched well with the standard data of CZTS (JCPDS no.26-0575). The main peaks corresponding to (112), (220), and (312) crystal planes of CZTS (Tao et al., 2015), which demonstrated that the CZTO precursor thin film was a suitable route in the preparation of CZTS thin film.

**Figure 11:**
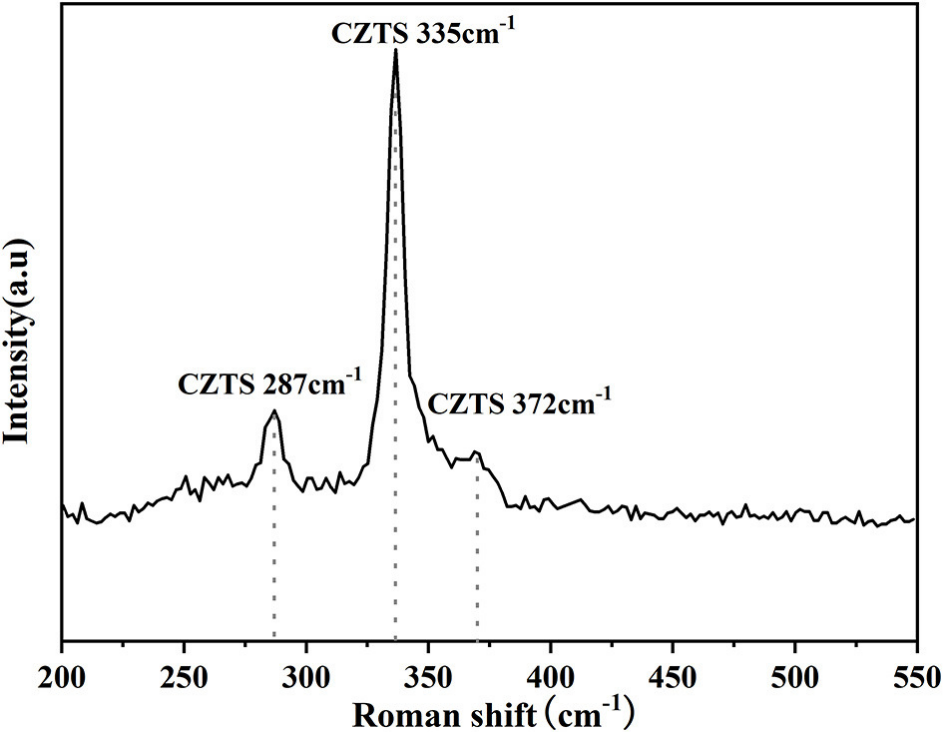
Raman analysis of CZTS thin film annealed at 580°C.

In general, the phase of CZTS, Cu_2_SnS_3_(CTS), and ZnS had very similar XRD diffraction patterns (Singh et al., 2012; Su et al., 2012). Therefore, Raman was measured to improve the phase differentiation. Figure 11 showed the Raman analysis of CZTS thin film annealed at 580°C. The sharp peaks could be observed at 287, 335, and 372 cm^−1^ (Scragg et al., 2014; Prabeesh et al., 2017; Wang et al., 2020) at 580°C proving the formation of pure-phase of Cu_2_ZnSnS_4_.

**Figure 12:**
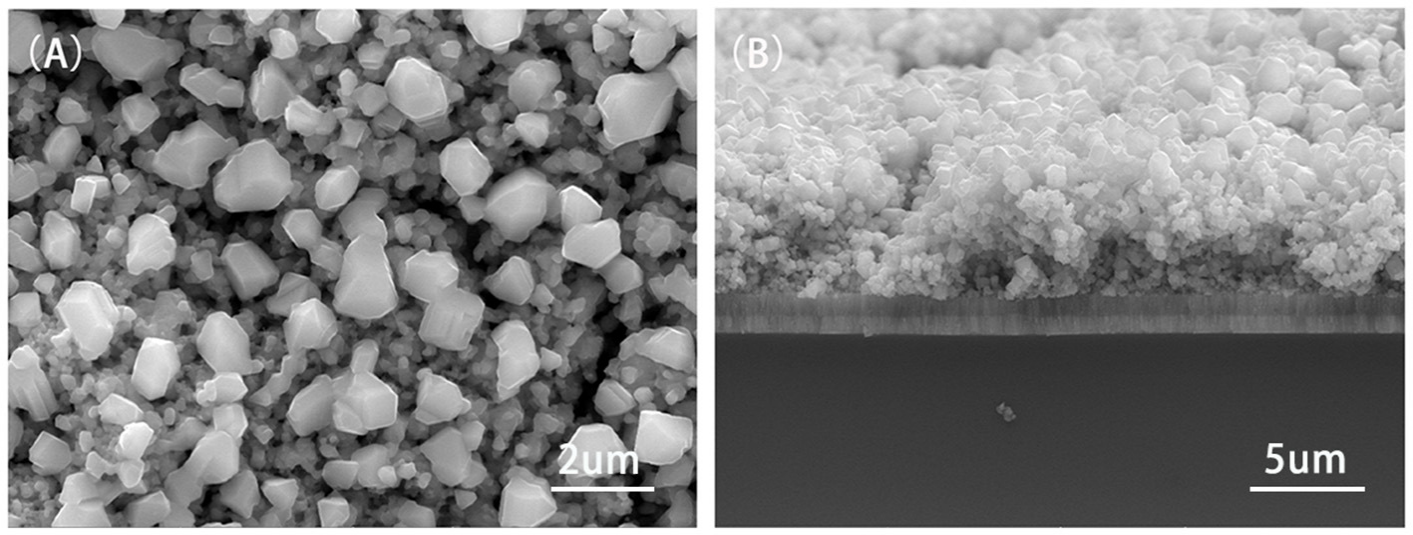
The surface **(A)** and cross-sectional **(B)** FESEM images of Cu_2_ZnSnS_4_ thin film annealed at 580°C.

Surface (A) and cross-sectional (B) views of Cu_2_ZnSnS_4_ thin film measured with FESEM annealing at 580°C were investigated in Figure 12. When the temperature reached 580°C, Cu_2_ZnSnS_4_ thin film showed a compact and uniform image with a size of about 1 μm, indicating that its crystalline quality was optimized. Cross section figures showed that the Cu_2_ZnSnS_4_ thin film was smooth. This would be ameliorated in the performance of CZTS solar cells owing to the improvement of crystal boundary and defects.

The electrical properties of Cu_2_ZnSnS_4_ thin film at 580°C, measured using the Hall effect method are presented in Table 2. The prepared CZTS thin film performed p-type behavior correspondence to some previous reports (Scragg et al., 2014). The value of the carrier concentration of CZTS thin film was in excess of 10^18^, which expressed the good electrical properties of CZTS thin film, confirming XRD and Raman measurements. The hall mobility of the CZTS thin film was 11.40 cm^2^/V·s, possibly because the large particles weakened the grain boundary, and also increased the lifetime of the carrier.

**Table 2:** Electrical-properties of CZTS film at 580°C.

**Carrier concentration(cm^**−3**^)**	**Hall mobility (cm^**2**^/V·s)**	**Resistivity(Ω·cm)**
8.894 × 10^18^	11.40	1.026

**Figure 13:**
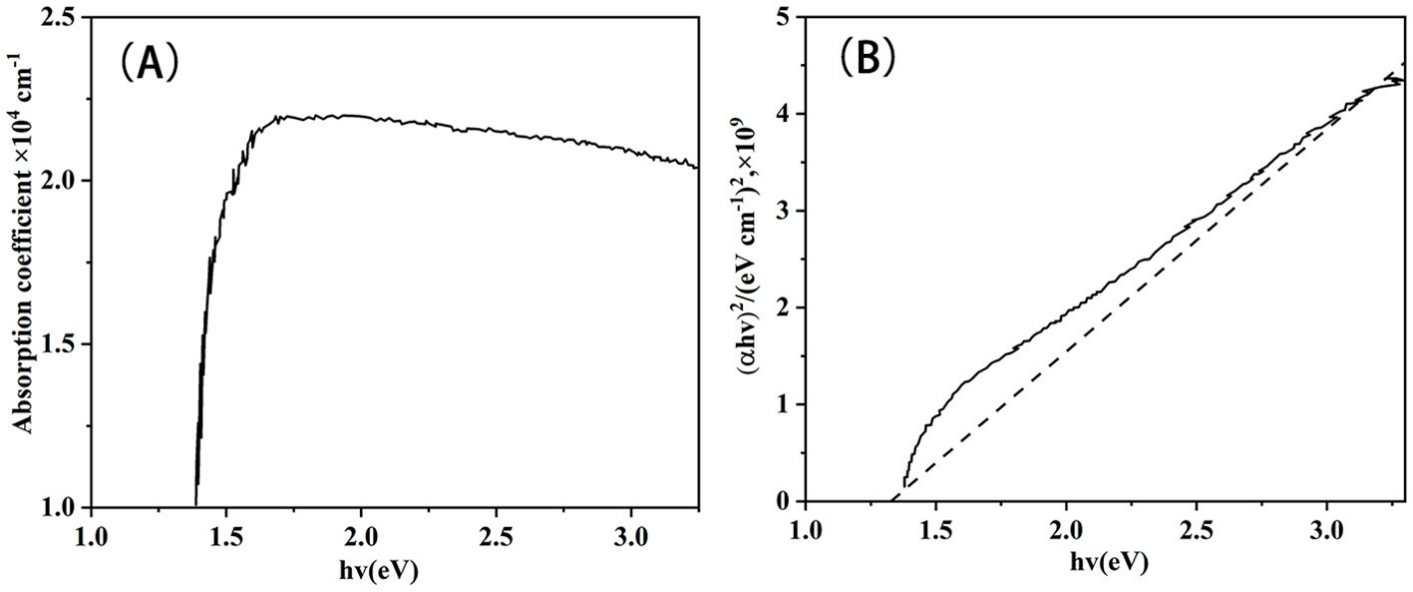
**(A)** Absorption spectra of CZTS thin film annealed at 580°C; **(B)** (αhv)^2^ vs. photon energy (hv) of CZTS thin film fabricated at 580°C.

The absorption coefficient (A) and (αhν)^2^ vs. hν of CZTS absorber layers (B) at 580°C tested by UV-Vis spectroscopy are shown in Figure 13. The optical band gap energy was determined to be 1.30 eV at 580°C, which is closed according to some previous reports (Wangperawong et al., 2011; Wang et al., 2012; Suryawanshi et al., 2016; Prabeesh et al., 2017). Therefore, the nano ink coating method was suitable for improving the performance of CZTS solar cells.

## Conclusion

In conclusion, the introduction of CZTO precursors can improve the uniform particle size and the dense surface of CZTS thin films, which effectively improves the surface defects and the quality of the thin films. By analyzing the effects of pH, co-precipitation temperature, and PVP on the composition and particle size of metal hydroxide, this study found that the co-precipitation temperature had a great effect on the composition of metal hydroxide. The ratios of Cu/(Zn+Sn) and Zn/Sn changed with temperature. When the temperature reached 40°C the ratios of Cu/(Zn+Sn) and Zn/Sn were 0.79 and 0.7, respectively, corresponding to the conditions for the preparation of CZTS thin films. Meanwhile, the 17 nm of particle size was conducive to the formation of dense thin films. In addition, when the pH was 9.5 and PVP content was 1%, metal hydroxide showed the suitable composition ratio and particle size. The effects of temperature and preservation time on the composition of CZTO calcinated by CZT hydroxides were further studied. The results showed that the CZTO obtained by calcination at 550°C for 1 h had the optimized composition and particle size. The influence of substrate spin temperature on the morphology of CZTO thin films was further investigated. The research proved that as spin temperature increased, the roughness of the CZTO surface decreased and the particle distribution became more uniform, profiting from the increased density of CZTS thin film formation. XRD curves showed that the CZTS thin films prepared by CZTO sulfurization at 580°C existed high peak strength and crystallinity, as well as reduced secondary phase, and corresponding well with standard CZTS cards (JCPDS no.26-0575). Raman curves proved that there were diffraction peaks of CZTS at 287, 335, and 372 cm^−1^, and no impurity peaks were found. According to the results, the CZTS thin films with hall mobility of 11.4 cm^2^/V·s, and the band gap of 1.30 eV were consistent with the theoretical band gap of CZTS, which was demonstrated that the CZTS thin film obtained by the sulfurization of CZTO had great electrochemical properties, and the simple nano ink coating method was also suitable for the improvement of CZTS solar cells.

## Data Availability Statement

The original contributions presented in the study are included in the article/Supplementary Material, further inquiries can be directed to the corresponding authors.

## Author Contributions

All authors listed have made a substantial, direct and intellectual contribution to the work, and approved it for publication.

## Conflict of Interest

The authors declare that the research was conducted in the absence of any commercial or financial relationships that could be construed as a potential conflict of interest.
