# Supplementary material for: Study on the Optimization of Cu-Zn-Sn-O to Prepare Cu2ZnSnS4 Thin Film via a Nano Ink Coating Method
Source: Front Chem. 2021 May 28;9:675642. doi: 10.3389/fchem.2021.675642 (PMC8195678; doi:10.3389/fchem.2021.675642)
Supplement: Supplementary file 1 [file Data_Sheet_1.ZIP › Tables.docx]

**TABLE 1** Compositions of precursor and Cu_2_ZnSnS_4_ by EDS

|  | Cu(at%) | Zn(at%) | Sn(at%) | O | S | Cu/(Zn+Sn) | Zn/Sn |
| --- | --- | --- | --- | --- | --- | --- | --- |
| Material | 50 | 25 | 25 | - | - | 1.00 | 1.00 |
| Precursor | 10.38 | 4.47 | 5.66 | 53.57 | - | 0.94 | 0.79 |
| Cu_2_ZnSnS_4_ | 22.95 | 11.38 | 12.51 | - | 53.16 | 0.96 | 0.91 |

**TABLE 2** Electrical-properties of CZTS film at 580°C

| Carrier concentration(cm^-3^) | Hall mobility (cm^2^/V·s) | Resistivity(Ω·cm) | Conductive type |
| --- | --- | --- | --- |
| 8.894×10^18^ | 11.40 | 1.026 | p |
